# Current and Historical Drivers of Landscape Genetic Structure Differ in Core and Peripheral Salamander Populations

**DOI:** 10.1371/journal.pone.0036769

**Published:** 2012-05-10

**Authors:** Rachael Y. Dudaniec, Stephen F. Spear, John S. Richardson, Andrew Storfer

**Affiliations:** 1 Department of Forest Sciences, University of British Columbia, Vancouver, British Columbia, Canada; 2 School of Biological Sciences, Washington State University, Pullman, Washington, United States of America; University of Lausanne, Switzerland

## Abstract

With predicted decreases in genetic diversity and greater genetic differentiation at range peripheries relative to their cores, it can be difficult to distinguish between the roles of current disturbance versus historic processes in shaping contemporary genetic patterns. To address this problem, we test for differences in historic demography and landscape genetic structure of coastal giant salamanders (*Dicamptodon tenebrosus*) in two core regions (Washington State, United States) versus the species' northern peripheral region (British Columbia, Canada) where the species is listed as threatened. Coalescent-based demographic simulations were consistent with a pattern of post-glacial range expansion, with both ancestral and current estimates of effective population size being much larger within the core region relative to the periphery. However, contrary to predictions of recent human-induced population decline in the less genetically diverse peripheral region, there was no genetic signature of population size change. Effects of current demographic processes on genetic structure were evident using a resistance-based landscape genetics approach. Among core populations, genetic structure was best explained by length of the growing season and isolation by resistance (i.e. a ‘flat’ landscape), but at the periphery, topography (slope and elevation) had the greatest influence on genetic structure. Although reduced genetic variation at the range periphery of *D. tenebrosus* appears to be largely the result of biogeographical history rather than recent impacts, our analyses suggest that inherent landscape features act to alter dispersal pathways uniquely in different parts of the species' geographic range, with implications for habitat management.

## Introduction

Processes structuring genetic diversity across species' ranges are complex, particularly as populations can vary in connectivity across heterogeneous or fragmented landscapes, or be influenced by geographically variable biogeographical histories that shape current genetic variation [1], [2]. The ‘central-marginal’ hypothesis predicts greater genetic diversity and gene flow toward the geographic centre of species' ranges, with less diversity and more genetic differentiation towards the distributional margins [3]–[5]. Recent studies indicate a variety of mechanisms that may shape diverse central-marginal genetic patterns (reviewed in [5]), such as historical processes occurring during post-glacial range expansion [6]–[8], long-distance dispersal events, biotic and abiotic events, and landscape heterogeneity [1]. Historical processes may result in genetic patterns that can be misinterpreted as effects of current anthropogenic disturbance at range margins [9]–[11], or conversely, show that current demographic processes override historical factors [4].

Peripheral populations generally occur in marginal habitats or areas that are climatically unfavourable, which may limit further expansion and result in genetically isolated populations that have an increased risk of local extinction [12]. The genetic underpinnings of these processes may result from founder events, with a decrease in effective population size (*N_e_*) and population connectivity at the periphery [3]. This becomes relevant for the evolutionary potential of species inhabiting peripheral habitats, and, is of particular concern for edge populations subject to fragmentation or climate change [8], [13], [14].

Hence, disentangling the effects of anthropogenic disturbance versus historical biogeographical processes across species' ranges will help to avoid bias in conservation strategies based on a single study area [7], [10], [15]. A combination of landscape genetics techniques and coalescent modelling provides a potential solution to this problem. In contrast to traditional methods that estimate gene flow with post-hoc inferences of landscape effects (e.g. [9], [16], [17]), landscape genetics can yield subtle quantitative differences in habitat or climatic variables that influence contemporary dispersal [18], [19]. Coalescent demographic modelling can be used to incorporate the phylogeographical framework in which a species' genetic diversity has been shaped [5]. For example, populations with a history of glaciation may have experienced large fluctuations in effective population size and migration rates that have shaped patterns of genetic variation in contemporary populations [5]. Together, landscape genetics and coalescent modelling offer a powerful means to test the relative influence of historical demography and contemporary landscape genetic patterns on genetic divergence and gene flow in central and peripheral parts of a species' range.

Amphibians are highly suited to the study of landscape genetic patterns due to their limited dispersal capacity and sensitivity to fine-scale landscape structure [20], [21]. In addition, amphibians are declining globally, highlighting the critical role of conservation strategies that are guided by molecular insights into habitat use and connectivity [22], [23]. Using the coastal giant salamander (*Dicamptodon tenebrosus*) as our focal species, we test whether historical or recent factors have affected population genetic structure at the species' northern periphery versus two core localities using a combination of coalescent modelling and a landscape genetics approach.

Our sample regions are located within the northern clade of *D. tenebrosus*, which was formed by post-Pleistocene range expansion from the Columbia River Valley in Washington State, USA, up to the northern range limit, which is delineated by the Fraser River in British Columbia [24], [25]. Post-glacial range expansions are expected to result in a reduction in *N_e_* and genetic variation at range margins [13]. In such ‘recently’ colonised areas, an overriding effect of historical factors is expected to result in reduced among-population genetic differentiation due to the homogenizing effect of continued gene flow and retained ancestral polymorphisms [4], [26], [27]. In contrast, a high degree of genetic structuring is suggestive of more recent factors that may be limiting dispersal, for example, recently formed landscape barriers or habitat fragmentation [9]. Highly active forestry activities throughout the range of *D. tenebrosus* over the last 100 years are a likely driver of fragmentation effects [16], [28], [29], [30].

Although not currently a species of concern in the United States, *D. tenebrosus* is listed as nationally ‘Threatened’ (COSEWIC, 2002) in Canada and is on the Provincial ‘Red List’ in British Columbia, primarily due to impacts of forest harvest and urban encroachment [16], [28], [30]. With over 75% of species at risk in Canada being at their northern range periphery, yet common in the continental USA, there is a need to distinguish inherent biological processes from anthropogenic disturbances that influence these populations, particularly under predicted pole-ward range shifts due to climate change [14].

Using a coalescent demographic simulation [31] we investigate changes in recent and historical *N_e_* in core and peripheral regions. Current influences on genetic structure are examined using a multiple pathway approach based on circuit theory that identifies how gene flow is limited by landscape resistance in terms of topographical, climatic and land cover features [2], [32]. This approach improves conventional gene flow models as it integrates all possible pathways connecting populations across the landscape [32].

Under expectations of the historical biogeography of *D. tenebrosus* [24] and central-marginal theory, we hypothesise that, (1) the peripheral region will have reduced genetic diversity and *Ne*, and if so, (2) historical range-expansion processes will have shaped current genetic patterns when the peripheral region shows a stable historic-to-recent population size compared with the core region, which is predicted to show an expansion signature (e.g. [26]), and, (3) recently formed landscape-driven genetic structure will be stronger at the periphery than the core if it is subject to increased stressors associated with habitat marginality or fragmentation. In addressing these hypotheses, we aim to tease apart whether factors impeding current gene flow are related to contemporary human impacts (e.g. deforestation, developed land) inherent landscape features (e.g. topography), and/or legacy effects of historical demographic processes within each region.

## Materials and Methods

### Ethics statement

All field and laboratory work was conducted with approval of the University of British Columbia Animal Care Committee (permit A08-0241) and the Washington State University Institutional Animal Care and Use Committee.

### Study System

The coastal giant salamander (*D. tenebrosus*) occurs in small streams from sea level to 1830 m elevation in the Pacific Northwest coniferous forests of the United States and south-western Canada [30], [33]. In Canada, the species occupies only a small area (∼100 km^2^) in the Chilliwack River watershed of British Columbia. *Dicamptodon tenebrosus* is highly sedentary [34] and has a gill-breathing larval stage lasting between 2–6 years prior to metamorphosis into the terrestrial adult form and reproductive maturity (at 15–35 cm total length) [30]. The species is believed to live for up to 20 years and is assumed to breed every two years [35]. It also shows facultative neoteny, whereby the larvae mature into gill-breathing, reproductive adults.

### Sampling

**Figure 1:**
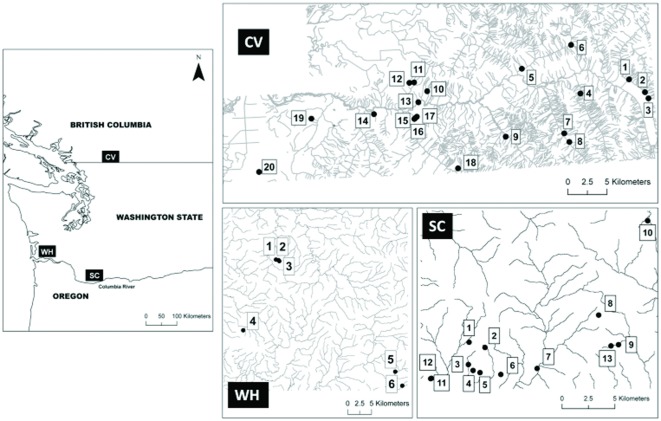
Map of three sampling regions in Washington State and British Columbia. WH = Willapa Hills; SC = South Cascades; CV = Chilliwack Valley. Site numbers correspond to those in Table S1. Some sites are located in small, unmarked streams.

*Dicamptodon tenebrosus* larvae, neotenes and terrestrial adults were sampled from a total of 39 randomly selected streams in three regions. Two core regions were selected in the United States within the southernmost area of the species' northern clade (extends through Washington State in to southern BC): Willapa Hills (WH), (area sampled ∼50 km^2^), South Cascades (SC) (area sampled ∼40 km^2^) (Figure 1). These sites were closed to the Pleistocene glacial refugium, which was inhabited by ancestral populations that formed the northern clade of *D. tenebrosus* [24]. Thus, these sites are expected to represent core populations with the highest genetic diversity. The third site was from the species' northernmost range in British Columbia, Canada, within the Chilliwack Valley (CV) (area sampled ∼70 km^2^ out of the 100 km^2^ total range in Canada) (Figure 1, Table S1). For WH and SC, sampling was conducted between March and September in 2006–2008 and for CV between June and August in 2008 and 2009. All individuals were sampled from 100–200 m transects within independent headwater streams (Figure 1, Table S1, described for CV in Dudaniec and Richardson [30]). A sample of tail tissue (2–10 mm^2^) was taken from each individual and preserved in 95% ethanol for DNA extraction as described in Steele et al. [36] and Dudaniec et al. [37].

### DNA extraction and genotyping

DNA extractions were performed using a standard phenol-chloroform ethanol precipitation protocol [38] or using a QIAGEN DNeasy 96 Tissue kit (QIAGEN, Inc.). Samples were genotyped at nine polymorphic microsatellite markers (Table S2) following conditions outlined in Steele et al. [39] and run on 96-well plates with negative and positive controls. For WH and SC samples, polymerase chain reaction (PCR) conditions for microsatellite amplifications followed those of Steele et al. [38]. Samples from CV followed PCR conditions described in Dudaniec et al. [37] for six loci. Products were genotyped on an ABI3730 automated sequencer (Applied Biosystems). The remaining three loci for CV (D04, D13 and D14) were amplified using a M13-tailed primer protocol [40] in a 10 µl PCR total reaction volume on a PTC-100 Thermocycler (MJ Research). PCRs contained 10X PCR Buffer, 2.0 mM dNTPs, 1.0 pmol each of M13-labelled forward and unlabelled reverse primer, 1.0 pmol M13- labelled reverse primer, 1 U Taq DNA polymerase, and 10–20 ng of genomic DNA. Amplification conditions followed those of Steele et al. [39] except denaturation, annealing, and elongation steps were increased to 60, 45 and 60 seconds respectively. For CV samples only, D04, D13 and D14 were genotyped on a LICOR sequencer with a 350 bp ladder and loci were scored manually using LI-COR SagaGT Software. Genotypes obtained from the LICOR sequencer were aligned with WH and SC data by subtracting the 18 bp M13 tail from all allele calls, and ten WH and SC samples were run on the LI-COR platform to confirm consistent allele size scoring across datasets. All other loci were manually scored using Genemapper 3.7 (Applied Biosystems, Inc.), and alleles were visually aligned to ensure consistent allele scoring across core and peripheral datasets (Table S2). All loci were scored by the same researcher (RYD).

Individuals with missing genetic data at three or more loci were excluded from the dataset. Individuals from each stream were screened for genetic relatedness in the program Colony 2.0 [41] and full sibs were removed from each stream, with one member of each full sib-ship retained. GenAlEx 6.2 [42] was used to obtain observed and expected heterozygosities for each locus. Each locus was tested for linkage disequilibrium and conformity to Hardy-Weinberg Equilibrium within each stream in Genepop 4.0.1 [43]. The presence of null alleles was assessed using MICROCHECKER 2.2.3 [44]. Significance was assessed following Bonferroni correction for multiple comparisons [45].

### Genetic differentiation between regions

Genetic differentiation between the three study regions (WH, SC, CV) was examined using six loci that were in HW equilibrium across all regions, which excluded D05, D17 and D25 (not in HW equilibrium for WH). Allelic richness within regions was calculated in FSTAT 2.9 [46] correcting for sample size. F_ST_ between regions and sites was calculated in Microsatellite Analyser 4.05 (MSA) [47] and significance assessed after Bonferroni correction (P<0.05). Partitioning of genetic variation within and across regions was examined using AMOVA in GenAlEx. To further confirm that gene flow between CV, WH and SC was restricted, and validate their classification as separate regions, we analysed all data in STRUCTURE [48]. Ten runs of *K* = 1–6 were performed with a 50 000 burn in and 1000000 Markov Chain Monte Carlo (MCMC) iterations, under a model of admixture and correlated allele frequencies. The number of genetic clusters (*K*) was determined using the method of Evanno et al. [49] and using the ln *K* method [50]. Individuals were assigned to clusters when the assignment probability was ≥0.7.

### Historical versus recent demographic processes

We examined whether there were historic or recent changes in effective population sizes and the timing of these changes in core versus peripheral regions, using MSVAR 1.3 [31], [51]. Given that SC contained the majority of genetic variation in the core regions, had a more comparable sample size with CV, and due to high computation requirements, we conducted separate simulations for SC and CV only. For consistency, we excluded locus D05 from CV (which was not in HW equilibrium within SC), resulting in an identical set of eight loci for each region. MSVAR 1.3 assumes a stepwise microsatellite mutation model [52] and estimates the posterior probability distribution of several parameters using Markov Chain Monte Carlo simulations based on the observed distribution of microsatellite alleles and their repeat numbers. The model assumes that the demographic parameters are identical across loci, while mutation rates are free to vary [31].

The parameters of interest for the current study were: current effective population size (*N_0_*), ancestral population size at the time of demographic change (*N_1_*), and the time in generations since population size change *T* = *T_a_*/*N_0_* (*T_a_* = number of generations since the beginning of the expansion/decline) [51]. Generation time is unknown for *D. tenebrosus*, yet we used a conservative generation time estimate of 12.5 years for simulations of ‘time since population change’, based on a predicted maximum life-span of 20 yrs [33]. The ratio of the posterior distributions of current and ancestral effective population sizes were calculated (where *r* = *N_0_*/*N_1_*) to determine population size changes where *r* = 1 indicates stability, *r*>1 indicates expansion, and *r*<1 indicates decline in the effective population size [31]. Stability of the estimates was evaluated by five independent simulations for both SC and CV, with a total number of 2×10^8^ updates and a thinning interval of 10 000 so that 20 000 estimated parameter sets were derived from the posterior distribution [31]. Each chain had a different starting value, and identical sets of starting values were used for each locus (Table S3). Wide parameter hyperpriors were applied to simulations, which varied slightly between runs to avoid possible effects on parameter estimates (Table S4).

We processed the output from MSVAR 1.3 using the program BOA 1.1.4 for R version 2.3.1 [53]. The first 10% of iterations were discarded from chains to avoid bias in parameter estimation where simulations may not have stabilised. Convergence of all chains was checked statistically using Brooks, Gelman and Rubin convergence diagnostic tests in BOA [54]. Convergence across chains is evident where the corrected scale-reduction factor approximates a value of 1, indicating the samples have arisen from a stationary distribution [53]. The potential scale-reduction factors for all three parameters were approximately 1 in SC, indicating convergence across chains (SC: *N_0_* = 1.00; *N_1_* = 1.08; *T* = 1.08) while in CV convergence was supported for *N_0_* (1.23) and *N_1_* (1.10), but less so for *T* (1.87). The last half of each chain was used to make a combined consensus chain of 50 000 data points, and summary statistics of the marginal posterior distributions for *N*_0_, *N*_1_ and *T* were estimated as the mean, 0.025 and 0.975 quantiles.

Girod et al. [55] showed that MSVAR was superior in its ability to detect population changes than the program Bottleneck [56], particularly with <10 loci, but performed poorly where recent bottlenecks have occurred. Therefore, we tested for deviation from mutation-drift equilibrium within streams using the program Bottleneck v1.2.02, which detects an excess or deficiency of heterozygotes relative to expected heterozygosity and is most appropriate for detecting recent bottlenecks [56]. We performed this analysis within each region, for each stream separately (WH = 6 loci, SC = 8 loci, CV = 9 loci). Both the two-phase (TPM) and step-wise (SMM) mutation models were used, with Wilcoxon sign-rank tests. The variance for the TPM was set at 5% and the proportion of SMM in TPM was set at 95% with 10 000 iterations [56]. We also examined for a mode shift distortion in the distribution of allele frequencies, whereby the loss of rare alleles during a recent bottleneck causes an increase in intermediate allele frequency classes [57].

### Genetic distances

Genetic differentiation (F_ST_) among streams within regions was calculated with Bonferroni correction using MSA. We compared the performance of two different measures of genetic distance between sample sites: G′_ST_ and D_ps_ (proportion of shared alleles averaged over loci). G′_ST_ is a standardized measure that is appropriate for examining genetic differentiation between datasets with different numbers of loci, and among loci with different levels of variation [58]. It also accounts for the high level of variability common in microsatellites, which can limit the upper bound of F_ST_ to be <1 [58]. We also conducted analyses with pairwise D_ps_ between sample sites (calculated in MSA) because this measure avoids equilibrium assumptions of G′_ST_ and is sensitive to genetic differences while controlling for low variation in allele frequencies among populations [59].

### Landscape data and resistances matrices

We chose landscape variables for analysis based on those shown to be important for *D. tenebrosus* occupancy, abundance or movement in previous studies, or those relevant to other stream-amphibians (described in Table 1). To evaluate the relative importance of landscape variables on genetic structure in *D. tenebrosus*, we modelled landscape resistances among sites using the program Circuitscape 2.2 [32], [60]. Circuitscape utilises circuit theory to evaluate the contribution of multiple pathways to the dispersal and gene flow of individuals according to landscape variables. Landscapes are represented as resistance surfaces, with user-defined low resistance habitats being more permeable to species movement than high resistance habitats. One focal point per site was identified and pairwise resistance matrices were calculated using the average resistance calculation under the four-node connection scheme.

**Table 1:** Mean (± s.e.) pairwise costs of dispersal between sites within each core and peripheral region for each landscape variable (Chilliwack Valley = CV; South Cascades = SC; Willapa Hills = WH).

Category	Variable (code)	Description/Categories	Ecological justification	Resistance direction	Periphery: CV (n = 20)	Core: SC (n = 13)	Core: WH (n = 6)
Topography	Isolation by resistance(IBR)	Flat landscape (all cells = 1) [60]	Equivalent to isolation by distance. Distance is a major feature limiting connectivity in amphibians [16], [78]	+	2.88 (0.1)	2.71 (0.1)	1.98 (0.2)
	Elevation(ELEV)	Elevation (m)	Connectivity can be reduced at high elevation streams [16], [30], [78]	+	1700.8 (53.7)	4843.0 (193.0)	3071.2 (300.1)
	Slope(SLP)	Steepness of study area (degrees)	Energetic costs of dispersal increases with slope [78], [79]	+	54.1 (1.0)	115.34 (3.4)	114.8 (8.6)
Habitat permeability	Canopy cover(CAN)	% of aerial canopy cover	Reduced cover is associated with dryness, higher temperatures and less species movement [34]	_	140.9 (3.0)	49.5 (1.3)	29.91 (4.1)
	Land cover(LC10; LC100)	1 = forest2 = non-forest natural*3 = barrier**	1- least cost to dispersal, 2- intermediate cost to dispersal, 3- highest cost to dispersal [61]	Cost ratios1 = *1;1*2 = *5; 50*3 = *10;100*	*1∶5∶10*4.26 (0.1)*1∶50∶100*5.4 (0.2)	*1∶5∶10*4.32 (0.1)*1∶50∶100*10.04 (0.9)	*1∶5∶10*4.07 (0.4)*1∶50∶100*5.5 (0.6)
	Stream vs other(STR10; STR100)	1 = stream cover2 = all other areas	Streams function as primary refuge and dispersal corridors due to favourable climate/resources [34], [36]	Cost ratios1 = *1;1*2 = *10;100*	*1∶10:* 20.1 (0.4)*1∶100:* 120.7 (3.7)	*1∶10:* 24.5 (0.8)*1∶100:* 202.1 (6.2)	*1∶10:* 30.1 (2.7)*1∶100:* 258.0 (19.6)
Temperature and precipitation	Frost-free period (growing season) (FFP)	Date of last freeze minus date of first freeze [62]	Longer periods without ice/snow may facilitate dispersal with decreased dispersal under longer FFP [20]	_	133.12 (4.5)	386.2 (18.0)	394.8 (48.7)
	Heat load index(HLI)	Measure of solar intercept [80]	Solar radiation decreases dispersal due to reduced thermal tolerance [20], [79]	+	2.8 (0.1)	2.5 (0.1)	5.22 (0.5)
	Growing season precipitation (GSP)	Rainfall during frost-free period (mm)	Wet areas favour dispersal and movement, which occurs during the growing season [30], [33], [77]	_	250.9 (7.0)	209.64 (8.6)	175.2 (23.6)

Ecological justification for each variable is presented with supporting references referring to *Dicamptodon* or other amphibians. The predicted direction of dispersal resistance for increasing values of each variable is indicated where ‘+’ = greater resistance, and ‘−’ = less resistance. n = sample size of populations.

* areas covered by non-forested, naturally occurring landscape features (e.g. shrub land, grass land, rock);

** barriers were defined as developed land, open water and perennial ice.

ArcGIS software version 9.3.1 (ESRI) was used to parameterise nine landscape variables, which pertain to topographic characteristics (elevation, slope), habitat permeability (canopy cover, landcover, stream vs. all other cover), or temperature and precipitation (frost-free period, heat load index, growing season precipitation) (described in Table 1; [20]). Isolation by resistance (IBR) matrices were calculated from raster layers with a ‘flat’ landscape (all cells with equal resistance of ‘1’) for each study region. IBR can be viewed as the equivalent of isolation by (log) Euclidean distance, but accounts for the finite size of the input landscape for each region, allowing its relative importance to other landscape variables to be assessed [60], [61]. All landscape variables had 30 m^2^ cell sizes (with the exception of frost-free period and growing-season precipitation which were at 750 m^2^ resolution) and each region was clipped with a minimum buffer of 500 m surrounding all sample sites to minimise ‘edge effects’ associated with calculating resistance values (as suggested by [62]).

Cell values for each landscape variable were converted directly into resistances based on expected linear predictions of suitability [3] (Table 1). For the categorical landscape variables (land cover and stream versus other cover), two different resistance ratios were analysed per variable to examine for variation in outcome (Table 1). Geographic data for CV were obtained from the GeoBase online resource for Canada: (http://www.geobase.ca/geobase/en/index.html (Canadian Council on Geomatics). For WH and SC, land cover and canopy cover data were from the 2001 National Land Cover dataset, stream data were from the National Hydrography Dataset (USGS 1999) and elevation, slope and heat load were derived from USGS digital elevation models. Frost-free period and growing season precipitation for both CV, WH and SC regions were estimated based on a spline model by Rehfeldt [63].

### Landscape genetic analysis

To test the relative effects of landscape variables on genetic distance we used multiple matrix regressions using the R statistics package (2.11.0). This analysis included resistance matrices of all landscape variables with genetic distance as the dependent variable in both regions (run separately for G′_ST_ and D_ps_). Akaike's Information Criterion (AIC) was applied separately to each region to find the best landscape model for explaining genetic distance between sites [64]. In accordance with Burnham and Anderson [65] multivariate models with the lowest change in AIC score (ΔAIC) and highest Akaike weights (ω) were considered the best models, models within two AIC units of these top models were regarded as interchangeable, and models within 10 units of the best value were interpreted as showing marginal support.

Nearly all variables were included in multiple models with substantial or moderate support (ΔAIC≤2); therefore we chose to use a model averaging approach. Model-averaged estimators often have a better measure of precision and reduced bias compared to estimators from just the selected best model [65]. To identify the combined effects of multiple landscape variables, we additively combined increasingly complex combinations of variables into multivariate landscape layers using ArcMap. We created multivariate landscapes by standardising all values for each landscape variable on a 1–10 scale (for every 30×30 m cell) and summing the standardised variables. Variables were selected for inclusion in multivariate models if their relative importance was ≥0.60 within the model averaged AIC result. We accounted for the effect of IBR by adding the IBR variable to every multivariate resistance model. Multivariate pairwise resistance matrices were created in Circuitscape 2.2 and AIC was applied to find the best model for each region. We performed correlation analysis on all variables for each region to aid in the interpretation of the results, such that highly correlated variables could be identified (Tables S5, S6, S7).

## Results

### Hardy-Weinberg and linkage equilibrium

After the exclusion of full-sibs (20–58%, mean = 38% of individuals collected per site), and individuals with >1/3 missing data (7% missing data for CV, 8% for WH, and 3% for SC), final sample sizes (n) were: CV, n = 387 (from 20 streams); WH, n = 213 (from 6 streams), and SC, n = 379 (from 13 streams) (Table S2). The number of individuals per stream available for analysis ranged from 10–86 (mean = 25.5±18.3 s.e.; Table S1). In CV, no loci were consistently out of HW equilibrium across sample sites. In WH, three loci (D05, D17 and D25) showed significant deviations from HW expectations and were excluded from the analysis, while D05 deviated from HW expectations in SC and was excluded from this region (Table S2). No loci were in linkage disequilibrium or showed evidence of null alleles after correcting for multiple comparisons.

### Genetic differentiation between regions

**Figure 2:**
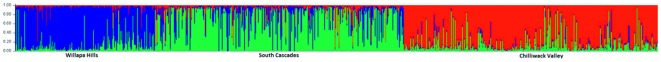
Assignment probability of each individual sampled from three regions. Three genetic clusters were identified (Willapa Hills, South Cascades, Chilliwack Valley) using the program STRUCTURE.

Genetic differentiation between WH, SC and CV was moderate and significantly different (Pairwise F_ST_: WH vs. SC = 0.04; SC vs. CV = 0.09; WH vs. CV = 0.16; P<0.02 all comparisons) (Tables S5, S6). AMOVA showed that 15% of the genetic variance was explained by region, 9% among streams, and 76% within streams. STRUCTURE consistently identified three genetic clusters corresponding to the three regions sampled, determined both by the ln *K* method and using the method of Evanno et al. [49] (Figure 2). The percentage of individuals correctly assigned to their source site with a probability of population membership ≥0.70 was 81.7% for WH (mean % = 0.91±0.005 s.e.), 61% for SC (mean % = 0.88±0.004 s.e.) and 83% for CV (mean % = 0.91±0.004 s.e.). Of those individuals assigned to a site other than their source site (174/251) 69% had assignment probabilities <0.70, which may indicate poor assignment power.

### Genetic diversity and differentiation within regions

Mean pairwise Euclidean distances (km ±s.e.) between sites for WH was 22.35 (±3.92), for SC was 9.5 (±0.75) and for CV was 17.6 (±0.8). Between core regions (WH+SC) pairwise F_ST_ = 0.04. Between SC and CV F_ST_ = 0.09, and for WH and CV F_ST_ = 0.16. All comparisons were significant after Bonferroni correction. Pairwise site F_ST_ comparisons were statistically different (P<0.05) after Bonferroni correction in 32.6% of comparisons for CV (Table S8), 37.5% for WH, and 34.6% for SC (Table S9). Values of pairwise F_ST_ (mean ±s.e.) between sites were moderate to low within regions (CV F_ST_ = 0.064±0.003; WH F_ST_ = 0.043±0.006; SC F_ST_ = 0.038±0.003). The peripheral region (CV) had lower allelic diversity in 7/9 loci (mean across all loci: 7.0±2.3 alleles per locus) compared to the core regions (WH: 11.8±5.1; SC: 13.7±6.4 alleles per locus) (Table S2). Allelic richness also showed decreased peripheral genetic diversity when correcting for sample size (n = 157) using six loci across all regions (WH = 11.67±2.15; SC = 12.48±2.60; CV = 6.14±2.40).

### Historical versus recent population decline

Coalescent-based simulations showed evidence for a ‘stable’ population in CV that exhibited virtually no detectable size change (*r* = 0.948), so *T* (time since change) could not be inferred (Table 2). Current effective population size in CV was *N_0_* = 419 (HPD interval: 448–4571), which was ∼33% lower than in SC, *N_0_* = 4286 (HPD interval: 904–19364). A slight historic population decline was detected in in SC (*r* = 0.802), estimated at approximately 849000 years ago (Table 2). Historical differences in population size were large, with a 94% smaller ancestral *N_e_* at the periphery compared to the core (Table 2).

**Table 2:** Results of MSVAR analysis assuming exponential change in population size.

Parameter	South Cascades (SC)		Chilliwack Valley (CV)	
	log_10_ scale	Converted value	log_10_ scale	Converted value
*T*	4.83 (±0.56) (3.74–5.88)	67920 (±3.63) (5546–763836)	not converged	not converged
*N_0_*	3.63 (±0.34) (2.96–4.29)	4286 (±2.18) (904–19364)	3.15 (±0.26) (2.65–3.66)	1419 (±1.81) (448–4571)
*N_1_*	4.544 (±0.41) (3.82–5.29)	34995 (±2.55) (6622–193196)	3.3 (±0.28) (2.79–3.87)	2138 (±1.88) (621–7379)

Values are presented as mean (± s.e.) on a log_10_ scale, and as converted values. Lower and upper bound Highest Probability Density intervals are within parentheses. *T* is the number of generations since population size change (runs did not converge for CV), *N_0_* = current and *N_1_* = ancestral effective population size.

There was no evidence for heterozygote excess in any core sites (Wilcoxon test: all P>0.05) although two sites in SC (15.4%) showed evidence of a mode shift in allele frequencies (Table S1). At the periphery, CV showed a heterozygote excess for 2/20 sites (10%, P<0.04), and an allele frequency mode shift in three sites. A significant heterozygote deficiency was found at the core for 5/13 sites (38.4%) in SC (P<0.04), 2/6 sites (33.3%) in WH (P<0.04), and for CV at the periphery, 4/20 (20%) sites (P<0.03) (Table S1). Results were consistent for both the TPM and SMM Wilcoxon tests.

### Landscape genetic structure at the core

No significant correlations were found between genetic distance and any landscape variable in the WH, including IBR, with AIC analysis showing that no models explained genetic structure better than a null model (all R^2^ = 0.00). Furthermore, all variables were highly correlated in WH (>0.80, Table S7), and no further analyses were conducted for this region. In SC, multiple matrix regressions with AIC model selection showed some support for nearly all variables using D_ps_ (averaged model R^2^ = 0.45). Variables with relative importance (RI) scores ≥0.60 were land cover (1∶5∶10 and 1∶50∶100 ratios, see Table 1), IBR, and frost-free period (Table 3), which were used for creating multivariate resistance surfaces for further analysis (Table 3). Multivariate landscape analyses revealed marginal support for an IBR relationship, but the strongest support was for the model combining IBR+frost-free period (R^2^ = 0.22) (Table 4). A two-variable model combining IBR, land cover, and frost-free period also had marginal support. Analyses using G′_ST_ were similar, with multiple matrix regression with AIC showing support for most models. RI values were ≥0.60 for IBR, frost-free period, canopy cover and land cover (1∶5∶10 and 1∶50∶100 ratios) (Table 3). Only land cover with a 1∶5∶10 resistance ratio was used in multivariate surfaces as it showed the highest RI. IBR had the strongest model support (R^2^ = 0.20), followed closely by IBR+frost-free period (R^2^ = 0.18, Table 4). All other models (except IBR+LC and IBR+CAN+LC) had marginal support. Although IBR and frost-free period were in the best models for both Dps and G′_ST_ in SC, IBR and FFP were highly correlated (0.99, Table S8), suggesting that the best G′ST model, consisting of just IBR, may best explain genetic distance in the core.

**Table 3:** Relative importance of landscape variables from multiple matrix regressions with AIC model selection.

		Relative importance (RI)			
Variable	Code	South Cascades (SC)		Chilliwack Valley (CV)	
		D_ps_	G′_ST_	D_ps_	G′_ST_
Isolation by resistance	**IBR**	**0.94**	**0.99**	0.29	0.51
Elevation	ELEV	0.43	0.37	**0.80**	0.49
Slope	SLP	0.47	0.51	0.53	**0.99**
Canopy cover	CAN	0.30	**0.78**	**0.64**	0.39
Frost-free period	FFP	**0.61**	**0.81**	0.51	0.46
Heat load index	HLI	0.37	0.33	**0.72**	**0.61**
Growing season precipitation	GSP	0.32	0.30	0.48	0.49
Land cover (1∶10)	LC10	**0.99**	**0.74**	0.50	0.34
Land cover (1∶100)	LC100	0.99	0.68	0.50	0.34
Stream vs. terrestrial (1∶10)	STR10	0.38	0.59	0.50	**0.60**
Stream vs. terrestrial (1∶100)	STR100	0.39	0.49	0.42	0.71

There were no results for the core Willapa Hills region due to the absence of a significant correlation of genetic distance with any landscape variable. Variables used for multivariate models (RI≥0.6) are in bold.

**Table 4:** Multivariate landscape models for explaining *D. tenebrosus* genetic structure in SC (South Cascades) and CV (Chilliwack Valley).

		D_ps_	AIC				G′_ST_	AIC			
Region	Model	Landscape features	R^2^	AIC	ΔAIC	ω	Landscape features	R^2^	AIC	ΔAIC	ω
SC	Isolation by resistance	*IBR*	*0.2*	*−178*	*1*	*0.3591*	**IBR**	**0.2**	**−98**	**0**	**0.5211**
		**IBR+FFP**	**0.22**	**−179**	**0**	**0.5920**	**IBR+FFP**	**0.18**	**−96**	**1**	**0.3160**
	Single variable	IBR+LC	0.02	−164	15	0.0003	IBR+LC	0.01	−84	13	0.0008
							*IBR+CAN*	*0.07*	*−88*	*10*	*0.0035*
	Two variable	*IBR+LC+FFP*	*0.15*	*−174*	*5*	*0.0486*	*IBR+LC+FFP*	*0.12*	*−92*	*6*	*0.0259*
							*IBR+FFP+CAN*	*0.16*	*−94*	*3*	*0.1163*
							IBR+CAN+LC	0.02	−84	14	0.0008
	Three variable	na	na	na	na	na	*IBR+FFP+CAN+ LC*	*0.11*	*−91*	*7*	*0.0157*
CV	Isolation by resistance	IBR	0.05	−537	16	0.0003	*IBR*	*0.05*	*−134*	*0*	*0.2094*
		**IBR+ELEV**	**0.13**	**−553**	**0**	**0.9416**	**IBR+SLP**	**0.05**	**−135**	**0**	**0.2094**
	Single variable	IBR+CAN	0.07	−541	12	0.0023	*IBR+STR*	*0.01*	*−128*	*6*	*0.0104*
		IBR+HLI	0.05	−536	17	0.0002	*IBR+HLI*	*0.05*	*−134*	*0*	*0.2094*
	Two variables	*IBR+ELEV+CAN*	*0.1*	*−547*	*6*	*0.0469*	**IBR+SLP+HLI**	**0.05**	**−135**	**0**	**0.2094**
		*IBR+ELEV+HLI*	*0.08*	*−543*	*10*	*0.0063*	*IBR+SLP+STR*	*0.03*	*−130*	*4*	*0.0283*
							*IBR +STR+ HLI*	*0.03*	*−131*	*4*	*0.0467*
	Three variables	IBR+ELEV+CAN+HLI	0.07	−541	12	0.0023	*IBR+SLP +STR+HLI*	*0.04*	*−132*	*2*	*0.0770*

Results of matrix regressions (model R^2^) and Akaike's Information Criterion (AIC, ΔAIC, and ω) are presented for G′_ST_ and D_ps_ measures of genetic distance. Models with the highest AIC support are in bold (i.e. within two units of the best model), and models with marginal support have italicised AIC values (i.e. within 10 units of the best model). na = not applicable. IBR = isolation by resistance; FFP = frost-free perod; LC = landcover; ELEV = elevation; CAN = Canopy cover; HLI = heat load index; STR = stream cover; SLP = slope.

### Landscape genetic structure at the periphery

Multiple matrix regression analysis for CV indicated some support for all variables using D_ps_ (overall models R^2^ = 0.24), but RI values ≥0.60 differed from SC, and included elevation, heat load index, and canopy cover (Table 3). Multivariate landscape analysis with D_ps_ showed that there was no independent effect of IBR, but that IBR+elevation best explained genetic distance (R^2^ = 0.13) (Table 4), and these two variables were not strongly correlated (0.31, Table S9). Two-variable models including elevation had marginal support. Analyses using G′_ST_ revealed support for most landscape variables (overall model R^2^ = 0.13) and RI values were ≥0.60 for slope, heat load index (0.71) and stream vs. other (1∶10 and 1∶100 ratios) (Table 3). RI was comparable for both resistance ratios for ‘stream vs. other’, therefore the 1∶10 resistance ratio was used in multivariate models. Multivariate landscape analysis with G′_ST_ showed the highest AIC scores and equal ω for IBR+slope and for IBR+slope+heat load index (Table 4). All other models showed marginal support (Table 4). However, heat load index was highly correlated with IBR (r = 0.99), but slope and IBR (r = 0. 34), and slope and heat load index (r = 0.41) were not, but we cannot rule out a combined influence of both variables (Table S9).

To summarise the main landscape genetic findings, our results for SC consistently showed an effect of IBR+FFP for D_ps_ and G′_ST_. However the high correlation between these variables means that the additional effect of frost-free period over simple isolation by resistance should be interpreted cautiously. However in CV, the topographical variables elevation (D_ps_) and slope (G′_ST_) clearly performed better than IBR alone, with the effect of solar radiation (i.e heat load index) being another possible factor influencing landscape genetic structure in the peripheral region.

## Discussion

By assessing only contemporary landscape genetic patterns, there is a risk of incorrectly attributing genetic patterns to recent landscape changes that are actually the result of historic biogeographical processes [11], [25], [66]. Our integration of both ‘historical’ demographic simulations and ‘recent’ landscape genetic analyses uncovers multiple drivers of population genetic structure within core and peripheral regions of *D. tenebrosus*. Historic range expansion effects appear to dominate current levels of genetic variation in both regions, with reduced *N*_e_ and genetic diversity at the periphery. Despite this, we reveal categorical differences in landscape effects on contemporary gene flow according to core or peripheral location, with stronger evidence for landscape-driven genetic structure at the periphery, in accordance with our hypotheses. Our results suggest that range-wide species conservation, may be better informed by concurrent analyses of historic demography and contemporary landscape genetic patterns that encompass more than one study area.

### Historical versus current demographic processes

In accordance with the ‘central-marginal’ hypothesis [1], [5], our coalescent simulations suggest that the northern periphery of *D. tenebrosus* had a much smaller founding population than the core, which is in accordance with the previously documented northward range expansion [24]. The low genetic diversity at the range periphery is consistent with previous genetic studies of *D. tenebrosus* conducted in the region [16], [37]. However, our genetic data did not support the prediction that anthropogenic disturbance has led to recent population decline at the threatened range periphery [16]. Rather, our coalescent analyses suggest that historical range expansion processes likely led to the observed reductions in genetic diversity, *N_e_*, and the current stable population signature at the periphery. Smaller populations may be more prone to extinction and have reduced adaptive potential, which can inhibit or slow range expansion into new environments, resulting in a stable population signature [67]. Despite apparent historical and recent population stability at the periphery, effects of recent processes shaping genetic structure were evident in the greater genetic differentiation among peripheral sites than among core sites. Furthermore, evidence of location-specific effects of landscape features on gene flow suggests likely dependence on demographic characteristics shaped by historic range expansion.

Whereas ancestral and current effective population sizes are much larger in the core, our analyses indicated a slight decline in *N_e_*, potentially due to a loss of genetic diversity during range expansion [1]. However, this signature should be interpreted cautiously as our samples were collected across a weakly structured population, which may result in a false bottleneck signal [68]. Despite this possibility, the time since population decline is consistent with previous estimations of separation dates for two refugia identified for *D. tenebrosus* within the early or mid-Pleistocene (1.7 mya ∼800000 ya) [24]. Our estimate of effective ancestral population size in SC of 34994 individuals accords with that of Steele and Storfer [24] who estimated *N_e_* to be 31563 in the Columbia River Valley refugium. In the core, current landscape effects on genetic structure were evident within SC, but there were no effects within WH. The strong correlations between landscape variables in WH (Table S7) further limited our ability to detect meaningful relationships, which may be a consequence of the reduced number of loci and sites sampled that influenced our ability to detect landscape genetic patterns. It is also possible that the extent of our study area was too small relative to the scale of genetic structure in WH, or the landscape was characterised by very low resistance [69].

Evidence for recent population bottlenecks was present in just 10% of peripheral sites with no evidence for bottlenecks in the core, where expansion signatures were evident in over 38% of sites. However, persistent population bottleneck signatures may not be detected where brief or even extreme population declines have occurred in the recent or distant past [15]. Our inability to detect recent bottlenecks in the core may also be attributed to low statistical power due to low sample sizes in some streams. Luikart and Cornuet [70] state that five to ten loci with 20–30 individuals should be effective to detect a recent bottlenecks using sign tests, but eight to ten loci is recommended for detecting a mode shift in allele frequency distribution with high probability [57]. Furthermore, *N*_e_ may be retained at substantial numbers despite a decline in census size due to high gene flow among local populations or across generations [4].

We conclude that although historic processes have largely created the underlying patterns of genetic variation across core and peripheral regions, current demographic processes continue to shape genetic structure.

### Landscape genetic patterns

Spatial replication in landscape genetic studies, both locally and regionally, is important for testing the generality of inferences about gene flow and landscape effects [71]. The differential core and peripheral landscape genetic patterns we found were not intuitively predictable based on mean differences in landscape variable resistance within each region. For example, mean resistance due to stream versus terrestrial cover and elevation were 40 and 60% lower, respectively, in the periphery than in the core (Table 1), but these variables were among the top models explaining peripheral resistance to dispersal. Additionally, solar radiation was comparable between regions yet was among the top models at the range periphery. This suggests that *D. tenebrosus* in CV has higher sensitivity to the landscape features we examined as compared to those in SC, and that the measured differences in landscape characteristics between regions do not necessarily predict the resulting landscape genetic relationships.

In small populations with low connectivity, we might expect greater landscape resistance according to topographic or land cover features. For large, genetically diverse populations, connectivity may be more influenced by broad-scale landscape variables (e.g. frost-free period, [20]) that represent a species' physiological or behavioural limitations. Our results show that the larger core population most strongly exhibits landscape genetic structure according to geographic distance (IBR) (though less clear due to correlation) and climatic tolerance (i.e. length of the growing season), rather than physical landscape features. In contrast, there was a dominant influence of topography (i.e. elevation and slope) on the strength of gene flow within the peripheral region independent of geographic distance, despite the core having approximately three times greater landscape resistance due to elevation and slope than the periphery (Table 1). Therefore, our results are not in accordance with expectations of resistance based on the differences in landscape structure between regions. This implies that multiple factors, such as population dynamics and genetic diversity, are strong drivers of landscape genetic patterns in addition to landscape features within a particular region.

Notably, Dudaniec and Richardson [30] show an increase in relative abundance of *D. tenebrosus* with site elevation (same sites sampled for the current study), indicating that census size does not equate to *Ne*/genetic diversity in these more productive populations.

Anthropogenic effects on peripheral landscape genetic structure were not detected explicitly (e.g. via the variables canopy cover or land cover), though solar radiation, which is postively related to forest harvest, was among the top models. However, Dudaniec and Richardson [30] show an increase in site relative abundance with time since forest harvest in the same sites sampled for this study. A temporal lag to detect a correlation between restricted gene flow and forest harvest effects may obscure our findings, as was the case for coastal tailed frogs in the Pacific Northwest (*Ascaphus truei*) [72].

### Encapsulating range-wide genetic patterns

Ecologically dissimilar habitats within a species' range can select for variation in adaptive traits that are likely to reflect landscape resistance to dispersal and genetic patterns [19], [73]. Also, differences in landscape genetic patterns may arise as the sample size of populations increases within an area as a result of greater genetic and spatial resolution of ecological processes [69]. Our sampling design enabled multiple spatial scales of genetic structure to be examined, with a wide range of distances between sites that are relevant to dispersal and genetic structure in *D. tenebrosus* [16], [36], [74]. Recent mixed ancestry for CV individuals with WH and SC is highly unlikely, given the ∼400 km distance from CV to WH and SC, coupled with the sedentary behaviour and low dispersal capability of *D. tenebrosus* [34],[74]. Although our results indicate some shared ancestry between WH and SC (∼150 km apart), our analyses provide strong evidence for three genetically distinct regions, validating their independent treatment.

The finding of greater genetic variation explained within streams than across regions (i.e. via AMOVA) may indicate non-equilibrium processes acting at different temporal and spatial scales, which can cause lower genetic differentiation between regions than that observed at lower hierarchical levels within regions (i.e. the stream level) [75], [76]. In *D. tenebrosus*, it can be expected that metapopulation processes may drive reductions in genetic variation and increased differentiation between streams within regions, while genetic variation at the regional level does not likely decline at the same rate.

From a spatial perspective, populations at ecological range limits may have a ‘patchier’ distribution, increasing pairwise genetic distances and landscape resistance. We acknowledge that the study area, and hence pairwise site distances were larger for CV than for SC and WH. However, previous studies suggest that a larger sample size and sampling effort is required in peripheral populations to capture the same proportion of genetic variation as in core populations due to stronger within-population spatial genetic structure [77]. Indeed, when controlling for sample size, patterns of allelic richness remained higher in the core than in the periphery, indicating little effect of sample size on our estimates.

Our results suggest that historic demography influences location-specific landscape genetic processes in core and peripheral populations of *D. tenebrosus*, but patterns are not consistent across regions with respect to the underlying differences in core and peripheral landscape characteristics. Although additional replicates of core and peripheral regions may help to resolve these disparate regional patterns, this lack of consistency suggests that historical demographic processes strongly influence our observed landscape genetic patterns. Although geologically recent colonisation has evidently shaped the lower genetic diversity at the periphery, these historical effects may act to exacerbate population sensitivity to habitat fragmentation resulting from forest harvest. Therefore, interactions between regional topography and anthropogenic disturbances should be considered for the conservation of threatened *D. tenebrosus* populations, and potentially other co-occurring, stream-associated amphibians. Our study demonstrates that combining both coalescent and landscape genetic analyses can help to disentangle current from historical processes that influence contemporary patterns of spatial genetic variability.

## Supporting Information

Table S1 Summary of collection sites (river drainage or label), site codes, final sample sizes (N) and geographical coordinates for British Columbia (BC) and Washington State (WA). Results from BOTTLENECK analyses are presented: ** indicates sites with significant heterozygote excess and a significant mode shift in allele frequency distribution; *indicates sites with just a significant mode shift in allele frequency distribution; † indicates sites with a significant heterozygote deficiency.
(DOC)

Table S2 Genetic diversity indices for each locus across all populations (N = number of populations; Na = number of alleles; Ho = mean observed heterozygosity; He = mean expected heterozygosity) and the number of populations (percentage in parentheses) not in Hardy-Weinberg equilibrium (HW) after correction for multiple comparisons (significance at the 0.05 level). Total sample sizes for each region are: Chilliwack Valley, n = 387, Willapa Hills, n = 213, South Cascades, n = 379. * locus excluded from analysis.
(DOCX)

Table S3 Parameter hyperpriors used for each simulation ran in MSVAR 1.3, for South Cascades and Chilliwack Valley datasets: ancestral population size (log N0), current population size (log N1), mutation rate (log u), and time since decline/expansion (log T).
(DOCX)

Table S4 Starting values for parameters for each locus used in all MSVAR simulations: ancestral population size (log N_0_), current population size (log N_1_), mutation rate (log u), and time since decline/expansion (log T). Starting values are the trial values for updating the parameters in the Metropolis-Hastings algorithm used in MSVAR. Locus D05 was excluded from the analysis for regional comparisons.
(DOCX)

Table S5 Pairwise Fst of *D. tenebrosus* between sampled streams in at the peripheral range in British Columbia, Canada: Chilliwack Valley (n = 387). Bold values were significantly different after Bonferroni correction.
(DOCX)

Table S6 Pairwise Fst of *D. tenebrosus* between sampled streams in Washington State, USA a) Willapa Hills, b) South Cascades. Bold values were significantly different after Bonferroni correction.
(DOCX)

Table S7 Correlation matrix (Pearson's r) of landscape variables for the Willapa Hills (WH) core region. STR10 = stream vs. terrestrial 1∶10, STR100 = stream vs. terrestrial 1∶100, LC10 = landcover 1∶10, CAN = canopy cover, FFP = frost free period, GSP = growing season precipitation, HLI = heat load index, IBR = isolation by resistance (flat), LC100 = landcover 1∶100, SLP = slope, ELEV = elevation.
(DOCX)

Table S8 Correlation matrix (Pearson's r) of landscape variables for the South Cascades (SC) core region. STR10 = stream vs. terrestrial 1∶10, STR100 = stream vs. terrestrial 1∶100, LC10 = landcover 1∶10, CAN = canopy cover, FFP = frost free period, GSP = growing season precipitation, HLI = heat load index, IBR = isolation by resistance (flat), LC100 = landcover 1∶100, SLP = slope, ELEV = elevation.
(DOCX)

Table S9 Correlation matrix (Pearson's r) of landscape variables for the Chilliwack Valley (CV) peripheral region. STR10 = stream vs. terrestrial 1∶10, STR100 = stream vs. terrestrial 1∶100, LC10 = landcover 1∶10, CAN = canopy cover, FFP = frost free period, GSP = growing season precipitation, HLI = heat load index, IBR = isolation by resistance (flat), LC100 = landcover 1∶100, SLP = slope, ELEV = elevation.
(DOCX)

## References

[1] Excoffier L, Foll M, Petit RJ (2009). Genetic consequences of range expansions.. Annual Review of Ecology, Evolution, and Systematics.

[2] Spear SF, Balkenhol N, Fortin M-J, McRae BH, Scribner KIM (2010). Use of resistance surfaces for landscape genetic studies: considerations for parameterization and analysis.. Molecular Ecology.

[3] Lesica P, Allendorf FW (1995). When are peripheral populations valuable for conservation?. Conservation Biology.

[4] Johansson M, Primmer CR, Merliä J (2006). History vs. current demography: explaining the genetic population structure of the common frog, *Rana temporaria*.. Molecular Ecology.

[5] Eckert CG, Samis KE, Lougheed SC (2008). Genetic variation across species' geographical ranges: the central–marginal hypothesis and beyond.. Molecular Ecology.

[6] Garner TWJ, Pearman PB, Angelone S (2004). Genetic diversity across a vertebrate species' range: a test of the central-peripheral hypothesis.. Molecular Ecology.

[7] Hoban S, Borkowski DS, Brosi SL, McCleary TL, Thompson LM (2010). Range-wide distribution of genetic diversity in the North American tree *Juglans cinerea*: a product of range shifts, not ecological marginality or recent population decline.. Molecular Ecology.

[8] Munwes I, Geffen E, Roll U, Friedmann A, Daya A (2010). The change in genetic diversity down the core-edge gradient in the eastern spadefoot toad (*Pelobates syriacus*).. Molecular Ecology.

[9] Jordan M, Morris D, Gibson S (2008). The influence of historical landscape change on genetic variation and population structure of a terrestrial salamander (*Plethodon cinereus*).. Conservation Genetics.

[10] Faurby S, King TL, Obst M, Hallerman EM, Pertoldi C (2010). Population dynamics of American horseshoe crabs – historic climatic events and recent anthropogenic pressures.. Molecular Ecology.

[11] Chiucci JE, Gibbs HL (2010). Similarity of contemporary and historical geneflow among highly fragmented populations of an endangered rattlesnake.. Molecular Ecology.

[12] Hoffmann AA, Blows MW (1994). Species borders: ecological and evolutionary perspectives.. Trends in Ecology and Evolution.

[13] Kawecki TJ (2008). Adaptation to marginal habitats.. Annual Review of Ecology, Evolution, and Systematics.

[14] Gibson SY, Van der Marel RC, Starzomski BM (2009). Climate change and conservation of leading-edge peripheral populations.. Conservation Biology.

[15] Okello JBA, Wittemyer G, Rasmussen HB, Arctander P, Nyakaana S (2008). Effective population size dynamics reveal impacts of historic climatic events and recent anthropogenic pressure in African elephants.. Molecular Ecology.

[16] Curtis JMR, Taylor EB (2003). The genetic structure of coastal giant salamanders (*Dicamptodon tenebrosus*) in a managed forest.. Biological Conservation.

[17] Dudaniec R, Gardner M, Donnellan S, Kleindorfer S (2008). Genetic variation in the invasive avian parasite, *Philornis downsi* (Diptera, Muscidae) on the Galapagos archipelago.. BMC Ecology.

[18] Manel S, Schwartz MK, Luikart G, Taberlet P (2003). Landscape genetics: combining landscape ecology and population genetics.. Trends in Ecology & Evolution.

[19] Storfer A, Murphy MA, Spear SF, Holderegger R, Waits LP (2010). Landscape genetics: where are we now?. Molecular Ecology.

[20] Murphy MA, Dezzani R, Pilliod DS, Storfer A (2010). Landscape genetics of high mountain frog metapopulations.. Molecular Ecology.

[21] Spear SF, Peterson CR, Matocq MD, Storfer A (2005). Landscape genetics of the blotched tiger salamander (*Ambystoma tigrinum melanostictum*).. Molecular Ecology.

[22] Storfer A (2003). Amphibian declines: future directions.. Diversity and Distributions.

[23] Stuart SN, Chanson JS, Cox NA, Young BE, Rodrigues ASL (2004). Status and trends of amphibian declines and extinctions worldwide.. Science.

[24] Steele CA, Storfer A (2006). Coalescent-based hypothesis testing supports multiple Pleistocene refugia in the Pacific Northwest for the Pacific giant salamander (*Dicamptodon tenebrosus*).. Molecular Ecology.

[25] Shafer ABA, Cullingham CI, Côté SD, Coltman DW (2010). Of glaciers and refugia: a decade of study sheds new light on the phylogeography of northwestern North America.. Molecular Ecology.

[26] Hewitt GM (1996). Some genetic consequences of ice ages, and their role in divergence and speciation.. Biological Journal of the Linnean Society.

[27] Bulgin JH, Stevens GC, Kaufman DM (1996). The geographic range: size, shape, boundaries, and internal structure.. Annual Review of Ecology and Systematics.

[28] Ferguson HM (1998). Demography, dispersal and colonisation of larvae of Pacific giant salamanders (*Dicamptodon tenebrosus*) at the northern extent of their range..

[29] Kroll AJ, Risenhoover K, McBride T, Beach E, Kernohan BJ (2008). Factors influencing stream occupancy and detection probability parameters of stream-associated amphibians in commercial forests of Oregon and Washington, USA.. Forest Ecology and Management.

[30] Dudaniec RY, Richardson JS Habitat associations of the Coastal Giant Salamander (*Dicamptodon tenebrosus*) at its threatened range limit..

[31] Beaumont MA (1999). Detecting population expansion and decline using microsatellites.. Genetics.

[32] McRae BH, Beier P (2007). Circuit theory predicts gene flow in plant and animal populations.. Proceedings of the National Academy of Sciences.

[33] Duellman W, Trueb L (1986). The biology amphibians..

[34] Ferguson HM (2000). Larval colonisation and recruitment in the Pacific giant salamander (*Dicamptodon tenebrosus*) in British Columbia.. Canadian Journal of Zoology.

[35] Nussbaum RA (1976). Geograpic variation and systematics of salamanders of the genus *Dicamptodon* Strauch (Ambystomatidae)..

[36] Steele CA, Baumsteiger J, Storfer A (2009). Influence of life-history variation on the genetic structure of two sympatric salamander taxa.. Molecular Ecology.

[37] Dudaniec RY, Storfer A, Spear SF, Richardson JS (2010). New microsatellite markers for examining genetic variation in peripheral and core populations of the coastal giant salamander (*Dicamptodon tenebrosus*).. PLoS ONE.

[38] Sambrook J, Russell DW (2001). Molecular cloning: A laboratory manual..

[39] Steele CA, Baumsteiger J, Storfer A (2008). Polymorphic tetranucleotide microsatellites for Cope's giant salamander (*Dicamptodon copei*) and Pacific giant salamander (*Dicamptodon tenebrosus*)..

[40] Schuelke M (2000). An economic method for the fluorescent labeling of PCR fragments.. Nature Biotechnology.

[41] Wang J (2004). Sibship reconstruction from genetic data with typing errors.. Genetics.

[42] Peakall R, Smouse PE (2006). Genalex 6: genetic analysis in Excel. Population genetic software for teaching and research.. Molecular Ecology Notes.

[43] Raymond M, Rousset F (1995). GENEPOP (Version 1.2): population genetics software for exact tests and ecumenicism.. J Heredity.

[44] van Oosterhout C, Hutchinson WF, Wills DPM, Shipley P (2004). MICRO-CHECKER: software for identifying and correcting genotyping errors in microsatellite data.. Molecular Ecology.

[45] Hochberg Y (1988). A sharper Bonferroni procedure for multiple tests of significance.. Biometrika.

[46] Goudet J (2001). FSTAT, a program to estimate and test gene diversities and fixation indexes (version.

[47] Dieringer D, Schlotterer C (2003). Microsatellite analyser (MSA): a platform independent analysis tool for large microsatellite data sets.. Mol Ecol Notes.

[48] Pritchard J, Stephens M, Donnelly P (2000). Inference of population structure using multilocus genotype data.. Genetics.

[49] Evanno G, Regnaut S, Goudet J (2005). Detecting the number of clusters of individuals using the software structure: a simulation study.. Molecular Ecology.

[50] Pritchard JK, Stephens M, Donnelly P (2000). Inference of population structure using multilocus genotype data.. Genetics.

[51] Storz JF, Beaumont MA (2002). Testing for genetic evidence of population expansion and contraction: an empirical analysis of microsatellite DNA variation using a hierarchical Bayesian model..

[52] Kimura M, Ohta T (1978). Stepwise mutation model and distribution of allelic frequencies in a finite population.. Proceedings of the National Academy of Sciences of the USA.

[53] Smith BJ (2005). BOA: Bayesian Output Analysis program..

[54] Brooks S, Gelman A (1998). General methods for monitoring convergence of iterative simulations.. Journal of Computational and Graphical Statistics.

[55] Girod C, Vitalis R, Leblois R, Fréville H (2011). Inferring population decline and expansion from microsatellite data: a simulation-based evaluation of the Msvar method.. Genetics.

[56] Cornuet JM, Luikart G (1996). Description and power analysis of two tests for detecting recent population bottlenecks from allele frequency data.. Genetics.

[57] Luikart G, Allendorf FW, Cornuet JM, Sherwin WB (1998). Distortion of allele frequency distributions provides a test for recent population bottlenecks.. Journal of Heredity.

[58] Hedrick PW (2005). A standardized genetic differentiation measure.. Evolution.

[59] Bowcock AM, Ruiz-Linares A, Tomfohrde J, Minch E, Kidd JR (1994). High resolution of human evolutionary trees with polymorphic microsatellites.. Nature.

[60] McRae BH (2006). Isolation by resistance.. Evolution.

[61] Lee-Yaw JA, Irwin JT, Green DM (2008). Postglacial range expansion from northern refugia by the wood frog, Rana sylvatica.. Molecular Ecology.

[62] Fortin M-J, Dale MRT (2005). Spatial analysis: a guide for ecologists..

[63] Rehfeldt GE (2006). A spline model of climate for the Western United States..

[64] Akaike H. Information theory as an extension of the maximum likelihood principle; 1973; Budapest..

[65] Burnham KP, Anderson DR (2002). Model selection and multi-model inference: a practical information theoretic approach..

[66] Zellmer AJ, Knowles L (2009). Disentangling the effects of historic vs. contemporary landscape structure on population genetic divergence.. Molecular Ecology.

[67] Bahn V, O' Connor RJ, Krohn WB (2006). Effects of dispersal at range edges on the structure of species' ranges..

[68] Chikhi L, Sousa VC, Luisi P, Goossens B, Beaumont MA (2010). The confounding effects of population structure, genetic diversity and the sampling scheme on the detection and quantification of population size changes.. Genetics.

[69] Anderson CD, Epperson BK, Fortin M-J, Holderegger R, James PMA (2010). Considering spatial and temporal scale in landscape-genetic studies of gene flow.. Molecular Ecology.

[70] Luikart G, Cornuet JM (1998). Empirical evaluation of a test for identifying recently bottlenecked populations from allele frequency data.. Conserv Biol.

[71] Short Bull RA, Cushman SA, Mace R, Chilton T, Kendall KC (2011). Why replicationis important in landscape genetics: American black bear in the Rocky Mountains..

[72] Spear SF, Storfer A (2008). Landscape genetic structure of coastal tailed frogs (*Ascaphus truei*) in protected vs. managed forests.. Molecular Ecology.

[73] Kark S, Lens L, Dongen V, Schmidt E (2004). Assymetry patterns across the distribution range: does the species matter?. Biological Journal of the Linnean Society.

[74] Johnston B, Frid L (2002). Clearcut logging restricts the movement of terrestrial Pacific giant salamanders (*Dicamptodon tenebrosus* Good).. Canadian Journal of Zoology.

[75] Pannell JR, Charlesworth B (2000). Effects of metapopulation processes on measures of genetic diversity.. Philosophical Transactions of the Royal Society B: Biological Sciences.

[76] Whitlock MC, McCauley DE (1999). Indirect measures of gene flow and migration: FST≠1/(4 Nm+1).. Heredity.

[77] Gapare WJ, Yanchuk AD, Aitken SN (2008). Optimal sampling strategies for capture of genetic diversity differ between core and peripheral populations of *Picea sitchensis* (Bong.) Carr.. Conservation Genetics.

[78] Giordano AR, Ridenhour BJ, Storfer A (2007). The influence of altitude and topography on genetic structure in the long-toed salamander (Ambystoma macrodactulym).. Molecular Ecology.

[79] Lowe WH, McPeek MA, Likens GE, Consentino BJ (2008). Linking movement behaviour to dispersal and divergence in plethodontid salamanders.. Molecular Ecology.

[80] McKune B, Keon D (2002). Equations for potential annual direct incident radiation and heat load.. Science.

