# Supplementary material for: Current and Historical Drivers of Landscape Genetic Structure Differ in Core and Peripheral Salamander Populations
Source: PLoS One. 2012 May 10;7(5):e36769. doi: 10.1371/journal.pone.0036769 (PMC3349670; doi:10.1371/journal.pone.0036769)
Supplement: Table S1 — Summary of collection sites (river drainage or label), site codes, final sample sizes (N) and geographical coordinates for British Columbia (BC) and Washington State (WA). Results from BOTTLENECK analyses are presented: ** indicates sites with significant heterozygote excess and a significant mode shift in allele frequency distribution; *indicates sites with just a significant mode shift in allele frequency distribution; † indicates sites with a significant heterozygote deficiency. (DOC) [file pone.0036769.s001.doc]

Table S1. Summary of collection sites (river drainage or label), site codes, final sample sizes (N) and geographical coordinates for British Columbia (BC) and Washington State (WA). Results from Bottleneck analyses are presented: ** indicates sites with significant heterozygote excess and a significant mode shift in allele frequency distribution; *indicates sites with just a significant mode shift in allele frequency distribution; † indicates sites with a significant heterozygote deficiency.

| **Drainage/stream** | **Site number** | **Site code** | **N** | **Latitude** | **Longitude** |
| --- | --- | --- | --- | --- | --- |
| **Chilliwack Valley (BC)**  Chilliwack Lake  Centre Creek  Foley Creek  Nesakwatch River  Slesse Creek  West Elk  Slesse Park  Little Tamihi  Tamihi North  Tamihi South  Vedder Mountain  Columbia Valley  **Willapa Hills (WA)**  Stream 1  Stream 2  Stream 3  Stream 4  Stream 5  Stream 6  **South Cascades (WA)**  Washougal River  Washougal River  Washougal River  Washougal River  Washougal River  Washougal River  Washougal River  Washougal River  Washougal River  Hamilton Creek  Gifford Pinchot Park  Stream A  Stream B | 1  2  3  4  5  6  7  8  9  10  11  12  13  14  15  16  17  18  19  20  1  2  3  4  5  6  1  2  3  4  5  6  7  8  9  10  11  12  13 | CL-1  CL-8*  CL-11  CCK  FOL-B  FOL-D†  NES-C  NES-5  SLC  WE-1  WE-2†  WE-4†  SP-1  LTAM  TAM-D†  TAM-E  TAM-F**  TAM-C**  VED  CV  3098  3110†  3111  3576  3914  5785†  01†  03  04  05  06  07*  08  10  11*  5378†  5595N†  5595S†  6000† | 29  17  13  10  13  30  10  31  21  16  28  16  10  26  25  26  18  12  32  18  17  38  74  10  44  30  12  17  16  18  18  20  21  13  10  21  51  76  86 | 0613728  0615686  0616094  0607431  0600100  0606520  0605150  0605755  0597653  0587884  0586264  0585657  0586689  0580961  0586234  0586427  0586095  0591397  0572952  0565978  443981  444517  444688  437121  468999  470472  561016  562526  560953  561395  562053  564021  567453  573280  575160  577925  557451  557388  574450 | 5438960  5437230  5436417  5437423  5440918  5443698  5432408  5431286  5432305  5438561  5439762  5439734  5437219  5435924  5435215  5435368  5435090  5428528  5435704  5429169  5159228  5159114  5158909  5144409  5135736  5132790  5061740  5061240  5059626  5059071  5058862  5058693  5059257  5064308  5061506  5073227  5058323  5058295  5061390 |
