# Supplementary material for: Current and Historical Drivers of Landscape Genetic Structure Differ in Core and Peripheral Salamander Populations
Source: PLoS One. 2012 May 10;7(5):e36769. doi: 10.1371/journal.pone.0036769 (PMC3349670; doi:10.1371/journal.pone.0036769)
Supplement: Table S2 — Genetic diversity indices for each locus across all populations (N = number of populations; Na = number of alleles; Ho = mean observed heterozygosity; He = mean expected heterozygosity) and the number of populations (percentage in parentheses) not in Hardy-Weinberg equilibrium (HW) after correction for multiple comparisons (significance at the 0.05 level). Total sample sizes for each region are: Chilliwack Valley, n = 387, Willapa Hills, n = 213, South Cascades, n = 379. * locus excluded from analysis. (DOCX) [file pone.0036769.s002.docx]

Table S2. Genetic diversity indices for each locus across all populations (N = number of populations; Na = number of alleles; Ho = mean observed heterozygosity; He = mean expected heterozygosity) and the number of populations (percentage in parentheses) not in Hardy-Weinberg equilibrium (HW) after correction for multiple comparisons (significance at the 0.05 level). Total sample sizes for each region are: Chilliwack Valley, n = 387, Willapa Hills, n = 213, South Cascades, n = 379. * locus excluded from analysis.

| **Location** | **Locus** | **Allele size range** | **Na** | **Ho** | **He** | **Number of populations not in HW (%)** |
| --- | --- | --- | --- | --- | --- | --- |
| Chilliwack Valley (N=20) | D04 | 152-184 | 8 | 0.354 | 0.371 | 0 (0) |
|  | D05 | 146-178 | 10 | 0.658 | 0.477 | 6 (30) |
|  | D07 | 271-315 | 4 | 0.753 | 0.483 | 7 (35) |
|  | D13 | 164-208 | 7 | 0.497 | 0.511 | 0 (0) |
|  | D14 | 157-173 | 5 | 0.399 | 0.362 | 0 (0) |
|  | D17 | 164-196 | 6 | 0.927 | 0.633 | 7 (35) |
|  | D18 | 206-246 | 11 | 0.408 | 0.568 | 2 (10) |
|  | D24 | 110-136 | 7 | 0.185 | 0.199 | 0 (0) |
|  | D25 | 101-117 | 5 | 0.167 | 0.175 | 1(5) |
| Willapa Hills (N=6) | D04 | 118-184 | 14 | 0.637 | 0.673 | 2 (33) |
|  | D05 | 167-230 | 16 | 0.325 | 0.711 | 5 (83)* |
|  | D07 | 259-315 | 4 | 0.209 | 0.289 | 1 (17) |
|  | D13 | 104-192 | 20 | 0.844 | 0.841 | 0 (0) |
|  | D14 | 87-173 | 13 | 0.694 | 0.671 | 1 (17) |
|  | D17 | 164-204 | 12 | 0.502 | 0.713 | 6 (100)* |
|  | D18 | 176-238 | 13 | 0.846 | 0.773 | 2 (33) |
|  | D24 | 120-148 | 9 | 0.255 | 0.268 | 0 (0) |
|  | D25 | 89-113 | 5 | 0.841 | 0.549 | 5 (83)* |
| South Cascades  (N=13) | D04 | 106-196 | 18 | 0.386 | 0.544 | 4 (31) |
|  | D05 | 147-215 | 13 | 0.267 | 0.688 | 13 (100)* |
|  | D07 | 271-315 | 4 | 0.456 | 0.462 | 1 (8) |
|  | D13 | 100-188 | 21 | 0.841 | 0.826 | 0 (0) |
|  | D14 | 91-187 | 22 | 0.756 | 0.721 | 0 (0) |
|  | D17 | 152-202 | 13 | 0.547 | 0.571 | 2 (15) |
|  | D18 | 136-250 | 17 | 0.833 | 0.785 | 2 (15) |
|  | D24 | 118-136 | 7 | 0.196 | 0.193 | 1 (8) |
|  | D25 | 85-113 | 8 | 0.284 | 0.282 | 1 (8) |
