# Supplementary material for: Current and Historical Drivers of Landscape Genetic Structure Differ in Core and Peripheral Salamander Populations
Source: PLoS One. 2012 May 10;7(5):e36769. doi: 10.1371/journal.pone.0036769 (PMC3349670; doi:10.1371/journal.pone.0036769)
Supplement: Table S3 — Parameter hyperpriors used for each simulation ran in MSVAR 1.3, for South Cascades and Chilliwack Valley datasets: ancestral population size (log N0), current population size (log N1), mutation rate (log u), and time since decline/expansion (log T). (DOCX) [file pone.0036769.s003.docx]

Table S3. Parameter hyperpriors used for each simulation ran in MSVAR 1.3, for South Cascades and Chilliwack Valley datasets: ancestral population size (log N_0_), current population size (log N_1_), mutation rate (log u), and time since decline/expansion (log T).

| **Parameter** | **Ancestral size** | | | | **Current size** | | | | **Mutation rate** | | | | **Time since decline/exp** | | | |
| --- | --- | --- | --- | --- | --- | --- | --- | --- | --- | --- | --- | --- | --- | --- | --- | --- |
| **Run** | **α** | **β** | **δ** | **τ** | **α** | **β** | **δ** | **τ** | **α** | **β** | **δ** | **τ** | **α** | **β** | **δ** | **τ** |
| **1** | 6 | 2 | 0 | 0.5 | 5 | 2 | 0 | 0.5 | -3.5 | 0.25 | 0 | 0.5 | 5 | 2 | 0 | 0.5 |
| **2** | 6 | 2 | 0 | 0.5 | 5 | 2 | 0 | 0.5 | -3.5 | 0.25 | 0 | 0.5 | 5 | 2 | 0 | 0.5 |
| **3** | 3 | 2 | 0 | 0.5 | 4 | 3 | 0 | 0.5 | -3.5 | 0.25 | 0 | 0.5 | 5 | 2 | 0 | 0.5 |
| **4** | 3 | 2 | 0 | 0.5 | 4 | 3 | 0 | 0.5 | -3.5 | 0.25 | 0 | 0.5 | 5 | 2 | 0 | 0.5 |
| **5** | 6 | 2 | 0 | 0.5 | 5 | 2 | 0 | 0.5 | -3.5 | 0.25 | 0 | 0.5 | 5 | 2 | 0 | 0.5 |
