# Supplementary material for: Current and Historical Drivers of Landscape Genetic Structure Differ in Core and Peripheral Salamander Populations
Source: PLoS One. 2012 May 10;7(5):e36769. doi: 10.1371/journal.pone.0036769 (PMC3349670; doi:10.1371/journal.pone.0036769)
Supplement: Table S4 — Starting values for parameters for each locus used in all MSVAR simulations: ancestral population size (log N0), current population size (log N1), mutation rate (log u), and time since decline/expansion (log T). Starting values are the trial values for updating the parameters in the Metropolis-Hastings algorithm used in MSVAR. Locus D05 was excluded from the analysis for regional comparisons. (DOCX) [file pone.0036769.s004.docx]

Table S4. Starting values for parameters for each locus used in all MSVAR simulations: ancestral population size (log N_0_), current population size (log N_1_), mutation rate (log u), and time since decline/expansion (log T). Starting values are the trial values for updating the parameters in the Metropolis-Hastings algorithm used in MSVAR. Locus D05 was excluded from the analysis for regional comparisons.

| **Locus** | ***Starting values*** | | | |
| --- | --- | --- | --- | --- |
|  | ***log N*_0_** | ***log N*_1_** | ***log* μ** | ***log* *T*** |
| **D04** | 1.0e4 | 1.0e4 | 1.0e-5 | 1.0e5 |
| **D07** | 5.0e4 | 1.0e4 | 2.0e-5 | 0.5e5 |
| **D13** | 1.0e4 | 1.0e4 | 0.1e-5 | 2.0e5 |
| **D14** | 1.0e4 | 1.0e4 | 1.0e-5 | 3.0e5 |
| **D17** | 1.0e4 | 5.0e4 | 2.0e-5 | 4.0e5 |
| **D18** | 1.0e4 | 1.0e4 | 3.0e-5 | 1.0e5 |
| **D24** | 1.0e4 | 1.0e4 | 0.5e-5 | 0.01e5 |
| **D25** | 1.0e4 | 1.0e4 | 0.5e-5 | 1.0e5 |
