# Supplementary material for: Current and Historical Drivers of Landscape Genetic Structure Differ in Core and Peripheral Salamander Populations
Source: PLoS One. 2012 May 10;7(5):e36769. doi: 10.1371/journal.pone.0036769 (PMC3349670; doi:10.1371/journal.pone.0036769)
Supplement: Table S5 — Pairwise Fst of D. tenebrosus between sampled streams in at the peripheral range in British Columbia, Canada: Chilliwack Valley (n = 387). Bold values were significantly different after Bonferroni correction. (DOCX) [file pone.0036769.s005.docx]

Table S5. Pairwise Fst of *D. tenebrosus* between sampled streams in at the peripheral range in British Columbia, Canada: Chilliwack Valley (n = 387). Bold values were significantly different after Bonferroni correction.

| **Fst** | **CCK** | **CL-1** | **CL-11** | **CV** | **FOL-B** | **FOL-D** | **LTAM** | **NES-5** | **NES-C** | **CL-8** | **SLC** | **VED** | **WE-1** | **TAM-D** | **TAM-E** | **TAM-F** | **WES-2** | **WE-4** | **TAM-C** |
| --- | --- | --- | --- | --- | --- | --- | --- | --- | --- | --- | --- | --- | --- | --- | --- | --- | --- | --- | --- |
| **CL-1** | 0.019 |  |  |  |  |  |  |  |  |  |  |  |  |  |  |  |  |  |  |
| **CL-11** | 0.189 | **0.141** |  |  |  |  |  |  |  |  |  |  |  |  |  |  |  |  |  |
| **CV** | 0.056 | **0.090** | **0.202** |  |  |  |  |  |  |  |  |  |  |  |  |  |  |  |  |
| **FOL-B** | 0.030 | 0.061 | 0.136 | 0.039 |  |  |  |  |  |  |  |  |  |  |  |  |  |  |  |
| **FOL-D** | 0.018 | **0.059** | **0.197** | 0.015 | 0.026 |  |  |  |  |  |  |  |  |  |  |  |  |  |  |
| **LTAM** | 0.052 | **0.066** | **0.175** | 0.031 | 0.062 | **0.042** |  |  |  |  |  |  |  |  |  |  |  |  |  |
| **NES-5** | 0.013 | **0.062** | **0.187** | **0.071** | 0.051 | 0.022 | **0.064** |  |  |  |  |  |  |  |  |  |  |  |  |
| **NES-C** | 0.021 | 0.068 | 0.117 | 0.065 | -0.010 | 0.043 | 0.077 | 0.043 |  |  |  |  |  |  |  |  |  |  |  |
| **CL-8** | 0.034 | 0.017 | -0.007 | **0.115** | **0.092** | **0.080** | 0.116 | 0.074 | 0.099 |  |  |  |  |  |  |  |  |  |  |
| **SLC** | 0.086 | **0.122** | **0.186** | 0.036 | **0.083** | **0.048** | **0.091** | **0.060** | **0.093** | **0.104** |  |  |  |  |  |  |  |  |  |
| **VED** | **0.091** | **0.111** | **0.183** | 0.024 | **0.070** | **0.044** | 0.043 | **0.070** | **0.088** | **0.117** | 0.016 |  |  |  |  |  |  |  |  |
| **WE-1** | 0.050 | **0.099** | **0.199** | **0.071** | 0.051 | **0.081** | **0.090** | **0.066** | 0.045 | 0.115 | **0.096** | **0.078** |  |  |  |  |  |  |  |
| **TAM-D** | 0.042 | **0.061** | **0.198** | 0.032 | 0.026 | 0.028 | 0.017 | **0.050** | 0.042 | 0.113 | **0.087** | **0.056** | 0.053 |  |  |  |  |  |  |
| **TAM-E** | 0.090 | **0.091** | **0.161** | 0.026 | 0.003 | **0.057** | 0.039 | **0.093** | 0.041 | **0.105** | **0.081** | 0.039 | 0.039 | 0.018 |  |  |  |  |  |
| **TAM-F** | 0.036 | **0.079** | **0.171** | 0.031 | 0.047 | **0.041** | 0.046 | **0.058** | 0.036 | **0.106** | 0.052 | 0.036 | 0.020 | 0.025 | 0.026 |  |  |  |  |
| **WE-2** | 0.003 | 0.065 | **0.155** | 0.032 | 0.062 | 0.025 | 0.057 | 0.024 | 0.054 | 0.063 | 0.026 | **0.046** | 0.050 | 0.060 | 0.086 | 0.002 |  |  |  |
| **WE-4** | 0.022 | 0.050 | **0.160** | 0.017 | -0.001 | 0.013 | 0.032 | 0.029 | 0.027 | 0.068 | 0.046 | 0.029 | 0.042 | 0.008 | 0.009 | 0.026 | 0.018 |  |  |
| **TAM-C** | 0.097 | 0.097 | **0.223** | -0.006 | 0.091 | 0.049 | 0.030 | **0.111** | 0.117 | 0.136 | 0.073 | 0.055 | 0.118 | 0.054 | 0.068 | 0.041 | 0.053 | 0.055 |  |
| **SP** | 0.045 | 0.051 | **0.181** | 0.010 | 0.054 | 0.017 | 0.028 | 0.035 | 0.072 | 0.052 | 0.023 | 0.037 | 0.081 | 0.041 | 0.061 | 0.027 | -0.012 | 0.007 | -0.005 |
