# Supplementary material for: Current and Historical Drivers of Landscape Genetic Structure Differ in Core and Peripheral Salamander Populations
Source: PLoS One. 2012 May 10;7(5):e36769. doi: 10.1371/journal.pone.0036769 (PMC3349670; doi:10.1371/journal.pone.0036769)
Supplement: Table S6 — Pairwise Fst of D. tenebrosus between sampled streams in Washington State, USA a) Willapa Hills, b) South Cascades. Bold values were significantly different after Bonferroni correction. (DOCX) [file pone.0036769.s006.docx]

Table S6. Pairwise Fst of *D. tenebrosus* between sampled streams in Washington State, USA a) Willapa Hills, b) South Cascades. Bold values were significantly different after Bonferroni correction.

| **Fst** | **3098** | **3110** | **3111** | **3576** | **3914** |
| --- | --- | --- | --- | --- | --- |
| **3110** | **0.031** |  |  |  |  |
| **3111** | **0.054** | **0.084** |  |  |  |
| **3576** | 0.026 | **0.083** | 0.035 |  |  |
| **3914** | 0.019 | **0.040** | **0.071** | 0.040 |  |
| **5785** | 0.015 | **0.044** | **0.052** | **0.041** | 0.011 |

a)

| **Fst** | **5378** | **5595N** | **5595S** | **6000** | **1** | **3** | **4** | **5** | **6** | **7** | **8** | **10** |
| --- | --- | --- | --- | --- | --- | --- | --- | --- | --- | --- | --- | --- |
| **5595N** | **0.051586** |  |  |  |  |  |  |  |  |  |  |  |
| **5595S** | **0.058907** | 0.010421 |  |  |  |  |  |  |  |  |  |  |
| **6000** | **0.039174** | **0.031092** | **0.013892** |  |  |  |  |  |  |  |  |  |
| **1** | **0.064023** | **0.065577** | 0.032462 | 0.025005 |  |  |  |  |  |  |  |  |
| **3** | **0.061671** | **0.065247** | **0.043725** | 0.024899 | 0.013819 |  |  |  |  |  |  |  |
| **4** | **0.081674** | **0.086286** | **0.062999** | 0.038907 | 0.006832 | 0.015353 |  |  |  |  |  |  |
| **5** | **0.093562** | **0.104364** | **0.06347** | 0.037404 | 0.024826 | 0.014796 | 0.022061 |  |  |  |  |  |
| **6** | 0.038394 | 0.032294 | 0.019084 | 0.011546 | 0.018457 | 0.008922 | 0.034423 | 0.010416 |  |  |  |  |
| **7** | 0.059716 | **0.063072** | **0.033857** | 0.015789 | -0.01771 | 0.002884 | 0.012105 | 0.015579 | 0.001104 |  |  |  |
| **8** | **0.062792** | **0.079211** | **0.051618** | 0.026076 | 0.010634 | 0.018542 | 0.029007 | 0.018132 | 0.004094 | -0.00507 |  |  |
| **10** | **0.073912** | **0.050012** | 0.023578 | 0.014825 | -0.00042 | 0.025563 | 0.026431 | 0.026459 | 0.014359 | 0.000701 | 0.005597 |  |
| **11** | **0.134026** | **0.101878** | 0.057963 | 0.05313 | 0.034194 | **0.098967** | **0.099973** | 0.063129 | 0.050209 | 0.025424 | 0.043538 | 0.016605 |

b)
