# Supplementary material for: Current and Historical Drivers of Landscape Genetic Structure Differ in Core and Peripheral Salamander Populations
Source: PLoS One. 2012 May 10;7(5):e36769. doi: 10.1371/journal.pone.0036769 (PMC3349670; doi:10.1371/journal.pone.0036769)
Supplement: Table S7 — Correlation matrix (Pearson's r) of landscape variables for the Willapa Hills (WH) core region. STR10 = stream vs. terrestrial 1∶10, STR100 = stream vs. terrestrial 1∶100, LC10 = landcover 1∶10, CAN = canopy cover, FFP = frost free period, GSP = growing season precipitation, HLI = heat load index, IBR = isolation by resistance (flat), LC100 = landcover 1∶100, SLP = slope, ELEV = elevation. (DOCX) [file pone.0036769.s007.docx]

Table S7. Correlation matrix (Pearson’s r) of landscape variables for the Willapa Hills (WH) core region. STR10 = stream vs. terrestrial 1:10, STR100 = stream vs. terrestrial 1:100, LC10 = landcover 1:10, CAN = canopy cover, FFP = frost free period, GSP = growing season precipitation, HLI = heat load index, IBR = isolation by resistance (flat), LC100 = landcover 1:100, SLP = slope, ELEV = elevation.

|  | STR10 | STR100 | LC10 | CAN | FFP | GSP | HLI | IBR | LC100 | SLP |
| --- | --- | --- | --- | --- | --- | --- | --- | --- | --- | --- |
| STR100 | 1 |  |  |  |  |  |  |  |  |  |
| LC10 | 1 | 1 |  |  |  |  |  |  |  |  |
| CAN | 0.97 | 0.98 | 0.97 |  |  |  |  |  |  |  |
| FFP | 0.99 | 0.98 | 0.99 | 0.96 |  |  |  |  |  |  |
| GSP | 0.99 | 0.98 | 0.99 | 0.98 | 0.99 |  |  |  |  |  |
| HLI | 0.99 | 0.99 | 1 | 0.98 | 0.99 | 0.99 |  |  |  |  |
| IBR | 0.89 | 0.86 | 0.89 | 0.82 | 0.94 | 0.91 | 0.89 |  |  |  |
| LC100 | 0.99 | 0.99 | 1 | 0.95 | 0.99 | 0.98 | 0.99 | 0.92 |  |  |
| SLP | 0.94 | 0.93 | 0.94 | 0.89 | 0.97 | 0.94 | 0.94 | 0.96 | 0.96 |  |
| ELEV | 0 | 0 | 0 | 0 | 0 | 0 | 0 | 0 | 0 | 0 |
