# Supplementary material for: Current and Historical Drivers of Landscape Genetic Structure Differ in Core and Peripheral Salamander Populations
Source: PLoS One. 2012 May 10;7(5):e36769. doi: 10.1371/journal.pone.0036769 (PMC3349670; doi:10.1371/journal.pone.0036769)
Supplement: Table S8 — Correlation matrix (Pearson's r) of landscape variables for the South Cascades (SC) core region. STR10 = stream vs. terrestrial 1∶10, STR100 = stream vs. terrestrial 1∶100, LC10 = landcover 1∶10, CAN = canopy cover, FFP = frost free period, GSP = growing season precipitation, HLI = heat load index, IBR = isolation by resistance (flat), LC100 = landcover 1∶100, SLP = slope, ELEV = elevation. (DOCX) [file pone.0036769.s008.docx]

Table S8. Correlation matrix (Pearson’s r) of landscape variables for the South Cascades (SC) core region. STR10 = stream vs. terrestrial 1:10, STR100 = stream vs. terrestrial 1:100, LC10 = landcover 1:10, CAN = canopy cover, FFP = frost free period, GSP = growing season precipitation, HLI = heat load index, IBR = isolation by resistance (flat), LC100 = landcover 1:100, SLP = slope, ELEV = elevation.

|  | STR10 | STR100 | LC10 | CAN | FFP | GSP | HLI | IBR | LC100 | SLP |
| --- | --- | --- | --- | --- | --- | --- | --- | --- | --- | --- |
| STR100 | 0.9069 |  |  |  |  |  |  |  |  |  |
| LC10 | 0.2953 | 0.1791 |  |  |  |  |  |  |  |  |
| CAN | 0.4928 | 0.2898 | 0.4899 |  |  |  |  |  |  |  |
| FFP | 0.9134 | 0.6846 | 0.2939 | 0.5987 |  |  |  |  |  |  |
| GSP | 0.8731 | 0.6279 | 0.3015 | 0.6185 | 0.9742 |  |  |  |  |  |
| HLI | 0.7584 | 0.6282 | 0.1105 | 0.5238 | 0.7734 | 0.7734 |  |  |  |  |
| IBR | 0.933 | 0.7178 | 0.3145 | 0.6242 | 0.9902 | 0.9666 | 0.8182 |  |  |  |
| LC100 | 0 | 0.007 | 0.5058 | 0 | 0.0162 | 0 | 0.0852 | 0 |  |  |
| SLP | 0.8201 | 0.6185 | 0.5282 | 0.6308 | 0.8525 | 0.8075 | 0.5096 | 0.853 | 0 |  |
| ELEV | 0.6956 | 0.4593 | 0.1415 | 0.4035 | 0.8587 | 0.8312 | 0.5034 | 0.7968 | 0.0332 | 0.6927 |
