# Supplementary material for: Current and Historical Drivers of Landscape Genetic Structure Differ in Core and Peripheral Salamander Populations
Source: PLoS One. 2012 May 10;7(5):e36769. doi: 10.1371/journal.pone.0036769 (PMC3349670; doi:10.1371/journal.pone.0036769)
Supplement: Table S9 — Correlation matrix (Pearson's r) of landscape variables for the Chilliwack Valley (CV) peripheral region. STR10 = stream vs. terrestrial 1∶10, STR100 = stream vs. terrestrial 1∶100, LC10 = landcover 1∶10, CAN = canopy cover, FFP = frost free period, GSP = growing season precipitation, HLI = heat load index, IBR = isolation by resistance (flat), LC100 = landcover 1∶100, SLP = slope, ELEV = elevation. (DOCX) [file pone.0036769.s009.docx]

Table S9. Correlation matrix (Pearson’s r) of landscape variables for the Chilliwack Valley (CV) peripheral region. STR10 = stream vs. terrestrial 1:10, STR100 = stream vs. terrestrial 1:100, LC10 = landcover 1:10, CAN = canopy cover, FFP = frost free period, GSP = growing season precipitation, HLI = heat load index, IBR = isolation by resistance (flat), LC100 = landcover 1:100, SLP = slope, ELEV = elevation.

|  | STR10 | STR100 | LC10 | CAN | FFP | GSP | HLI | IBR | LC100 | SLP |
| --- | --- | --- | --- | --- | --- | --- | --- | --- | --- | --- |
| STR100 | 0.8283 |  |  |  |  |  |  |  |  |  |
| LC10 | 0.5093 | 0.2365 |  |  |  |  |  |  |  |  |
| CAN | 0.6377 | 0.3079 | 0.9062 |  |  |  |  |  |  |  |
| FFP | 0.2207 | 0.0374 | 0.4747 | 0.537 |  |  |  |  |  |  |
| GSP | 0.4361 | 0.1261 | 0.5436 | 0.6848 | 0.7894 |  |  |  |  |  |
| HLI | 0.7502 | 0.3676 | 0.7144 | 0.8124 | 0.4682 | 0.7021 |  |  |  |  |
| IBR | 0.6344 | 0.3279 | 0.5172 | 0.6477 | 0.3922 | 0.5951 | 0.7623 |  |  |  |
| LC100 | 0.3014 | 0.1224 | 0.9347 | 0.7621 | 0.4306 | 0.3811 | 0.4826 | 0.3294 |  |  |
| SLP | 0.3667 | 0.1883 | 0.4483 | 0.5108 | 0.5596 | 0.4396 | 0.4126 | 0.3443 | 0.3978 |  |
| ELEV | 0.1826 | 0.0352 | 0.4329 | 0.4648 | 0.9677 | 0.6687 | 0.376 | 0.3125 | 0.4213 | 0.5588 |
